# GLIM criteria represent a more suitable tool to evaluate the nutritional status and predict postoperative motor functional recovery of older patients with hip fracture: A retrospective study

**DOI:** 10.1097/MD.0000000000037128

**Published:** 2024-02-09

**Authors:** Weicheng Wu, Zhening Guo, Zenghui Gu, Yongtao Mao, Chang She, Jun Gu, Bo Lv, Wei Xu, Liubing Li

**Affiliations:** a Department of Orthopedics, The Second Affiliated Hospital of Soochow University, Suzhou, China; b Department of Pharmacy, The Second Affiliated Hospital of Soochow University, Suzhou, China; c State Key Laboratory of Radiation Medicine and Protection, Soochow University, Suzhou, China.

**Keywords:** GLIM criteria, hip fracture, malnutrition, MNA-SF, NRS-2002, postoperative functional recovery

## Abstract

Early recognition of malnutrition is essential to improve the prognosis of older patients with hip fracture. The Nutritional Risk Screening 2002 (NRS-2002), the Short-Form Mini Nutritional Assessment (MNA-SF) and the Global Leadership Initiative on Malnutrition (GLIM) are widely used in malnutrition diagnosis. However, criteria for predicting postoperative hip joint motor function in older patients with hip fractures are still necessary. The objective of this study was to select the most appropriate criteria from the NRS-2002, the MNA-SF and the GLIM in predicting the postoperative hip joint motor function recovery 1 year after surgery. This retrospective observational study included 161 patients aged ≥ 65 years with hip fractures. The nutritional status of patients was determined by the NRS-2002, MNA-SF and GLIM. The Harris hip joint score (HHS), the primary outcome of this study, was used to evaluate hip joint motor function. HHS was classified as excellent (HHS > 75) or non-excellent outcomes (HHS ≤ 75). Logistic regression models for hip joint motor function recovery were constructed. Both the receiver operating characteristic curve and the decision curve analysis were used to select the most predictive criteria. The overall mean age of the 161 patients was 77.90 ± 8.17. As a result, NRS-2002 (OR:0.06, 95%CI [0.01, 0.17]), MNA-SF (OR:0.05, 95%CI [0.00, 0.23]) and GLIM (OR of moderate: 0.03, 95%CI [0.01, 0.11]; OR of severe: 0.02 [0.00, 0.07]) were predictive for recovery of hip joint motor function. Additionally, both the area under curve of the receiver operating characteristic curve (NRS-2002: 81.2 [73.8, 88.6], MNA-SF: 76.3 [68.5, 84.2], GLIM: 86.2 [79.6,92.8]) and the decision curve analysis showed the GLIM was better than others. Compared with NRS-2002 and MNA-SF, GLIM was a more suitable nutritional assessment criteria to predict the postoperative recovery of hip joint motor function for older patients with hip fracture 1 year after surgery.

## 1. Introduction

Hip fracture is the most severe form of osteoporotic fracture, and with the older people, the global incidence of hip fractures is projected to rise from 1.26 million in 1990 to 4.5 million by 2050.^[1]^ Due to its high cost, high mortality rate, substantial disability rate, and various complications,^[2,3]^ hip fracture has become a global public health problem.^[4]^

As previous studies shown, 14% to 65% of patients with hip fracture are malnourished,^[5]^ which increases the incidence of postoperative complications, prolongs the length of hospital stay, and increases mortality. Especially, many malnourished hip fracture patients suffer from loss of functional independence and affects their long-term motor functional recovery after surgery.^[2,6,7]^ Some studies have found that timely nutritional assessment and intervention measures for patients with malnutrition can effectively improve the prognosis.^[8]^ Therefore, it is important to quickly and accurately evaluate the nutritional status in older patients with hip fracture.

To determine the nutritional status, some nonspecific serum biomarkers such as albumin and prealbumin were used to evaluate the nutritional status and predict prognosis in patients with hip fracture.^[9]^ However, these biomarkers are not recommend to identify malnutrition due to they are not only affected by nutritional status, but by many other factors such as infection and liver injury.^[10]^ Instead, nutritional screening tools are commonly used in clinical practice to identify patients at risk of malnutrition now, which include Nutrition Risk Screening 2002 (NRS-2002), the Short-Form Mini Nutritional Assessment (MNA-SF).^[11–13]^ The NRS-2002 is a widely used nutritional screening tool that has been validated to effectively predict the prognosis of hospitalized patients.^[14]^ Moreover, MNA-SF is designed specifically for the older people and is predictive of motor functional status at discharge during the acute phase with hip fracture patients.^[6]^ However, these standards have their own shortcomings, and there is still no globally recognized gold standard for diagnosis of malnutrition. For example, MNA-SF shows high sensitivity but low specificity often overestimates the nutritional risk of older patients.^[15]^ Although there are many kinds of nutritional screening tools, it is still unclear which tools were more suitable for the older patients with hip fracture.

The Global Leadership Initiative on Malnutrition (GLIM) has gradually reached a consensus on clinical diagnosis of malnutrition. The GLIM criteria feature a secondary assessment of patients assessed by a nutritional screening tool to better assess their nutritional status. For patients at risk of malnutrition according to nutritional screening, the GLIM criteria recommend nutritional assessment, including phenotypic indicators (involuntary weight loss, low body mass index (BMI) and muscle mass loss), and etiological indicators (reducing food intake/inflammation and disease burden). To diagnose malnutrition, there should be at least 1 phenotypic and 1 etiological index. After the diagnosis of malnutrition, the GLIM criteria classify the degree of malnutrition into moderate or severe.^[16]^ GLIM criteria have been applied to gastrointestinal diseases, tumors and cardiovascular diseases,^[17–19]^ and the prognosis of patients is well predicted.

Given that hip fracture among older people is associated with high reduction of patients’ motor function, we compared the GLIM criteria with MNA-SF and NRS-2002 commonly used in clinical practice, and to select the most appropriate nutritional screening tool in evaluating malnutrition and postoperative motor functional recovery 1 year after surgery.

## 2. Material and methods

### 2.1. Study population

**Figure 1.**
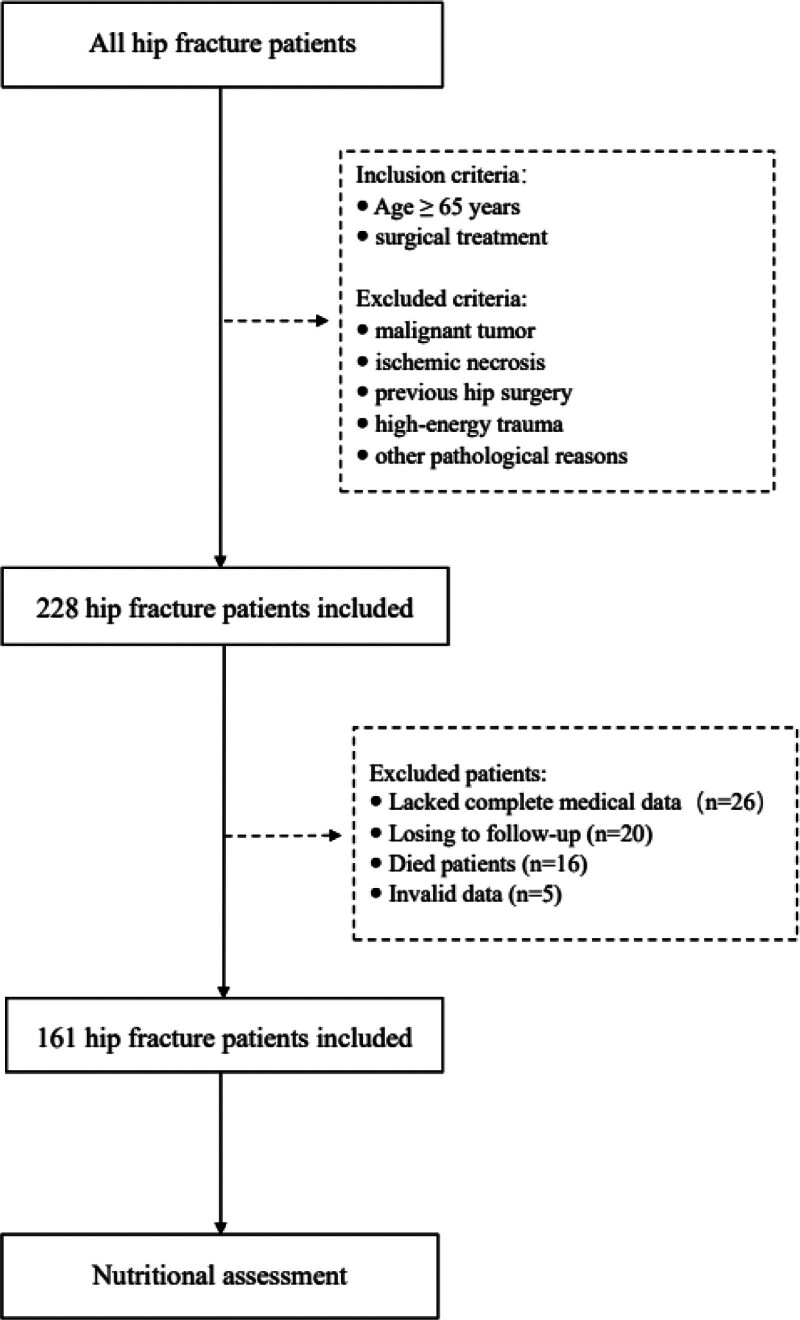
A flow chart of the screening for the study. GLIM = global leadership initiative on malnutrition; MNA-SF = mini nutrition Assessment; NRS-2002 = Nutrition Risk Screening 2002.

This study retrospectively analyzed patients with hip fracture who underwent surgery in the Second Hospital of Soochow University between January and December 2021. The patients were all over 65 years old, and the surgery performed included hip replacement and internal fixation. We excluded patients with fracture due to malignant tumor, ischemic necrosis, previous hip surgery, high-energy trauma, and other pathological reasons. Patients with missing indicators were also excluded. A flow chart of the study screening is shown in Figure 1. The study was approved by the Medical Ethics Committee of the Second Hospital of Soochow University.

### 2.2. Data collection

We collected demographic and clinicopathological data, including sex, age, fracture type, height, weight, CRP and BMI. We also used Harris hip joint score (HHS) to evaluate the motor functional results of patients 1 year after surgery through telephone follow-up. HHS is a standardized assessment of patients’ hip with a maximum of 100 points, which consisted of further subscales with greater weight on function as assessed by activities of daily living and gait (47 points) and pain (44 points), and less weight on absence of deformity (4 points) and range of motion (5 points). This scoring system have been previously validated for use in our patient population after hip arthroplasty.^[20]^ In the current study, HHS > 75 was defined as better recovery and HHS ≤ 75 was defined as bad recovery.

### 2.3. Nutritional assessment

All patients were initially screened with NRS-2002 (patients with a score ≥ 3 were considered to be at risk of malnutrition) and MNA-SF (patients with a score ≤ 11 were considered to be at risk of malnutrition). After screening by NRS-2002 and MNA-SF respectively, the malnutrition patients assessed by GLIM criteria and the analysis results showed no significant difference. Finally, for patients with NRS-2002 scores ≥ 3, we proceeded to the second step of diagnosis using the GLIM criteria.

#### 2.3.1. Nutritional Risk Screening 2002.

The NRS-2002 system is a two-step screening procedure with an initial evaluation of BMI, weight loss during the last 3 months, reduced dietary intake during the last week, and severe disease.^[21]^ The score is age-adjusted and ranges from 0 to 7. Patients with a value ≥ 3 were assigned to the malnourished group and a value < 3 indicated the normal nutrition group.

#### 2.3.2. Mini nutritional assessment-short form.

Mini nutritional assessment-short form the MNA-SF tool consists of the following parameters: food intake, weight loss, mobility, psychological stress, neuropsychological problems, and BMI. All parameters are scored from 0 to 2 or 3 with a total score of 0 to 14.^[12]^ Patients with a total score. Patients with a total score ≤ 11 were classified as malnourished while a total score >11 was classified as normal nutrition.

#### 2.3.3. The global leadership initiative on malnutrition.

The GLIM criteria were used to evaluate the diagnosis and severity of malnutrition and were composed of 3 phenotypic and 2 etiological criteria. The combination of at least 1 phenotypic and 1 etiological index was a necessary condition for the diagnosis of malnutrition.^[22]^ The details of the GLIM are as follows^[17]^: (1) weight loss: a nonvolitional weight loss of 5% to 10% within the past 6 months, or 10% to 20% beyond 6 months was defined as moderate malnutrition. A nonvolitional weight loss of > 10% within the past 6 months, or > 20% prior to the past 6 months was considered to indicate severe malnutrition. (2) BMI cutoffs for malnutrition risk were < 20 kg/m2 if < 70 years, and < 22 kg/m^2^ if ≥ 70 years, which was defined as moderate malnutrition. BMI of < 18.5 kg/m2 for those aged < 70 years, and < 20 kg/m^2^ for those aged ≥ 70 years, was considered to severe malnutrition. (3) Reduced muscle mass: the muscle mass was estimated by the appendicular skeletal muscle mass (ASM) using a previously validated equation in a Chinese population: ASM = 0.193 × weight (kg) + 0.107 × height (cm)— −4.157 × sex (male = 1, female = 2)— −0.037 × age (year) −2.631. The ASM equation model is in good agreement with the DXA (adjusted R2 = 0.90, standard error of estimate = 1.63 kg). After estimating the ASM values, the skeletal muscle mass index (SMI) was calculated using the ASM divided by the square of the height in meters (SMI = ASM/height^[2]^).^[23,24]^ SMI < 7.00 kg/m^2^ in men and < 5.70 kg/m^2^ in women which was defined as moderate malnutrition. SMI < 5.20 kg/m^2^ in men and < 4.70 kg/m^2^ in women which was defined severe malnutrition.^[25,26]^ (4) Reduced food intake or absorption was defined as an intake of 50% or less of energy requirements for > 1 week, or any reduction for > 2 weeks. (5) Increased disease burden or inflammation: chronic or recurrent mild-to-moderate inflammation was likely to be associated with malignant disease or any disease that was considered chronic or recurrent. Presence of inflammation was defined as CRP ≥ 5 mg/L.^[27]^

### 2.4. Statistical analysis

All statistical analyses were performed using R software (version 4.0.2). The quantitative variables were expressed as mean ± SD, and the categorical variables as count (percentage). Logistic regression was used to construct both univariable and multivariable nutritional screening models. The decision curve analysis (DCA) and area under the receiver operating characteristic curve (ROC) (area under curve [AUC]) were used to evaluate the performance of prediction on motor functional recovery. The bootstrap method was used to construct the calibration curve to validate prediction of motor functional recovery. *P* < .05 was statistically significant.

## 3. Results

### 3.1. Clinicopathological characteristics

In the primary cohort, there were initially 228 patients, excluding those who lost contact, died and had missing data All patients were evaluated by MNA-SF, NRS-2002, and GLIM criteria, people who are defined as malnutrition are included in the follow-up study. A total of 161 patients who met the inclusion criteria were finally enrolled in the study (Fig. 1). There were 107 women (66%) and 54 men (34%), with an average age of 77.90 ± 8.17 years. The demographic features and clinical characteristics of the primary and validation cohorts are presented in Table 1.

**Table 1:** Clinical characteristics of hip fracture patients.

GLIM criteria	Normal	Moderate	Severe	Overall
n	94	43	24	161
Age	75.71 ± 7.40	79.26 ± 8.21	84.04 ± 7.62	77.90 ± 8.17
*Gender*				
Male	37 (39.4%)	14 (32.6%)	3 (12.6%)	54 (33.5%)
Female	75 (60.6%)	29 (67.4%)	21 (87.4%)	107 (66.5%)
Height	1.60 ± 0.08	1.61 ± 0.08	1.55 ± 0.08	1.60 ± 0.08
Weight	62.59 ± 9.23	52.45 ± 5.76	43.08 ± 5.54	56.98 ± 10.75
BMI	24.26 ± 2.70	20.22 ± 1.88	17.80 ± 1.38	22.22 ± 3.45
*Fracture type*				
ITF	39 (41.5%)	13 (30.2%)	10 (41.7%)	62 (38.5%)
FNF	55 (58.5%)	30 (69.8%)	14 (58.3%)	91 (61.5%)
*Surgery type*				
Replacement	55 (58.5%)	29 (67.4%)	11 (45.8%)	95
Fixation	39 (41.5%)	14 (32.6%)	13 (54.2%)	66
NRS-2002 Risk (%)	17 (18.1%)	43 (100.0%)	24 (100.0%)	84 (54.7%)
MNA-SF Risk (%)	48 (51.1%)	41 (100.0%)	24 (100.0%)	113 (70.7%)

BMI = body mass index; FNF = femoral neck fracture; ITF = intertrochanteric fracture; MNA-SF = Mini Nutrition Assessment; NRS-2002 = Nutrition Risk Screening 2002.

### 3.2. GLIM-defined malnutrition

Among the 161 older patients with hip fracture, according to the GLIM criteria, there were 67 patients (41.6%) with malnutrition, including 43 (26.7%) with moderate malnutrition and 24 (14.9%) with severe malnutrition (Table 1).

### 3.3. Comparison of motor functional recovery of patients with GLIM, MNA-SF and NRS-2002 criteria

According to HHS, the patients were divided into better recovery of postoperative motor function (HHS > 75) and worse recovery of postoperative motor function (HHS ≤ 75). According to the univariable model, the postoperative motor function of patients was significantly associated with age(OR:0.93, 95%CI [0.89, 0.97]), GLIM criteria(OR of moderate: 0.03, 95%CI [0.01, 0.10]; OR of severe: 0.02, 95%CI [0.00, 0.06]), MNA-SF (OR:0.03, 95%CI [0.01, 0.10]) and NRS-2002 (OR:0.05, 95%CI [0.01, 0.15]) (*P* < .05) (Table 2). After controlling for gender and age, multivariable logistic regression models were performed on the 3 nutritional screening tools. The results showed that NRS-2002 (OR:0.06, 95%CI [0.01, 0.17]), MNA-SF (OR:0.05, 95%CI [0.00, 0.23]) and GLIM (OR of moderate: 0.03, 95%CI [0.01, 0.11]; OR of severe: 0.02 [0.00, 0.07]) could effectively predict the recovery of postoperative motor function (Table 2).

**Table 2:** Univariable and multivariable logistic regression models.

	Univariable model	Multivariable models		
		NRS-2002	MNA-SF	GLIM
Gender (Male)	2.13 [0.096, 5.12]			
Age	0.93 [0.89, 0.97]*	0.96 [0.91, 1.01]	0.95 [0.90, 0.99]*	0.98 [0.93, 1.04]
*Fracture type*				
FNF	Reference			
ITF	0.85 [0.41, 1.76]			
*Surgery type*				
Replacement	Reference			
Fixation	0.85 [0.42, 1.76]			
NRS-2002	0.05 [0.01, 0.15]*	0.06 [0.01, 0.17]**		
MNA-SF	0.04 [0.00, 0.19]*		0.05 [0.00, 0.23]*	
*GLIM*				
Normal	Reference			
Moderate	0.03 [0.01, 0.10]**			0.03 [0.01, 0.11]**
Severe	0.02 [0.00, 0.06]**			0.02 [0.00, 0.07]**

FNF = femoral neck fracture; GLIM = global leadership initiative on malnutrition; ITF = intertrochanteric fracture; MNA-SF = Mini Nutrition Assessment; NRS-2002 = nutrition risk screening 2002.

* *P* < .05.

** *P* < .01.

**Figure 2.**
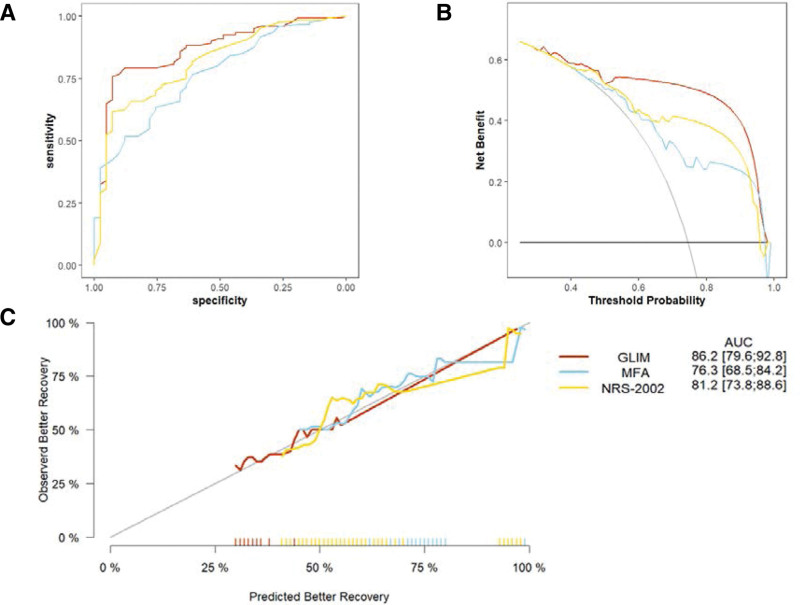
(A) Receiver operating characteristic curves of the GLIM, MNA-SF, and NRS-2002 models. (B) Decision curve analysis of the GLIM, MNA-SF, and NRS-2002 models. (C) Risk calibration curve for predicting postoperative recovery. GLIM, global leadership initiative on malnutrition; MNA-SF = mini nutrition assessment;NRS-2002 = Nutrition Risk Screening 2002.

AUC of the ROC curve of the GLIM model was 86.2, which was larger than that of the NRS-2002 (AUC = 81.2) and MNA-SF (AUC = 76.3) (Fig. 2A). We found that the GLIM criteria were better able to predict prognosis of older patients with hip fracture. According to the decision curve analysis, the 3 nutritional screening models all showed predictive effect on postoperative recovery (Fig. 2B). However, GLIM model has higher net benefit than that of the other 2 models. The calibration curve was constructed by bootstrap method (Fig. 2C), in which the apparent and biased-corrected curve were both close to the ideal line. The Hosmer–Lemeshow test also showed acceptable fitness of the model (χ^2^ = 5.0952, *P* = .7474).

### 3.4. Validation of the predictive accuracy of the nomogram

**Figure 3.**
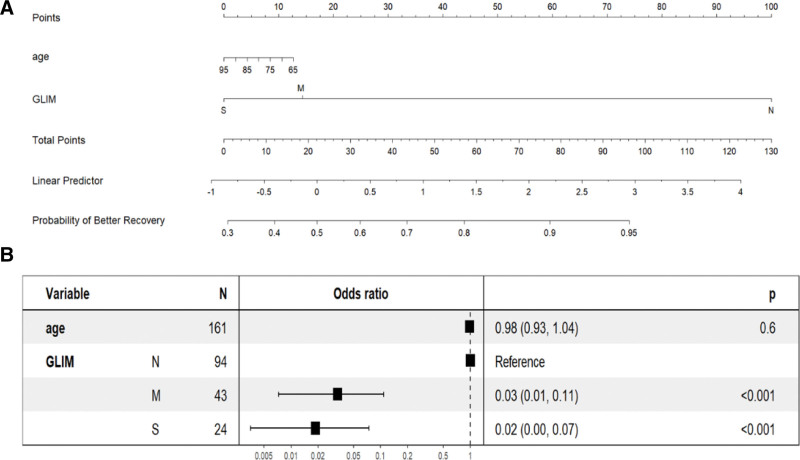
(A) The nomogram developed to predict postoperative recovery of older hip fracture patients at risk of malnutrition. (B) The forest plot based on global leadership initiative on malnutrition domains and clinical variables of older hip fracture patients.

The GLIM model was selected as the best to predict motor functional recovery, and the scores of the predictors were coded and shown in the nomogram in Figure 3A. As shown in the Forest plot (Fig. 3B), GLIM criteria defined malnutrition severity revealed affecting postoperative motor functional recovery significantly, in which OR of moderate level and severe level were 0.03 (0.01, 0.11) and 0.02 (0.00, 0.07) respectively.

## 4. Discussion

Malnutrition is common among older patients with hip fracture, and is a major global public health problem. Nutritional status affects the prognosis of older patients, and timely nutritional assessment is helpful for treatment. This study used the GLIM criteria of the global consensus on the diagnosis of malnutrition to retrospectively analyze patients who underwent surgery for hip fracture. GLIM criteria has proven to be an effective way to assess nutritional status for many diseases since it published. Camilla et al proved that GLIM criteria is more sensitive than other nutritional screening tools for inflammatory bowel disease.^[28]^ Gazcon-Ruiz et al pointed out that the GLIM criteria for malnutrition in patients with head and neck tumors have a higher sensitivity than the European Society for Clinical Nutrition and Metabolism criteria.^[29]^ Through this study, we found that the GLIM standard can be used to predict postoperative motor function recovery in patients with hip fracture, and have a better predictive value than MNA-SF and NRS-2002.

We collected data for 161 patients, for whom the prevalence of malnutrition was 41.6%, including 26.7% with moderate malnutrition and 14.9% with severe malnutrition. The prevalence ate of malnutrition in patients with hip fracture diagnosed by different nutrition screening tools in the study of Inoue et al was 7% to 26%.^[30]^ The prevalence of malnutrition measured by MNA-SF in patients with hip fracture in the study of Malafarina et al was 18.7%.^[2]^ However, if other diagnostic criteria (BMI, albumin or weight loss) are used, the prevalence rate was higher (45.7%).^[2]^ In the study of Gaek et al study, the prevalence rate of malnutrition in patients screened by MUST is was 36.2%.^[15]^ The results of our study showed that the incidence of malnutrition was not significantly different from that of previous studies.

Hip fracture often leads to the reduction of older patients’ motor function and quality of life.^[31]^ Most patients cannot recover their quality of life and motor function even after surgery. According to Li et al, malnourished patients with hip fracture after surgery have significantly worse performance trajectories for their activities of daily living, instrumental activities of daily living, and recovery of walking ability.^[32]^ Previous studies used nutritional screening tools such as MNA-SF which confirmed that nutritional status affects the motor functional recovery of patients with hip fracture after surgery.^[6,7,33]^ Timely improvement of patients’ nutritional status can improve motor functional recovery after surgery.^[34]^ However, there is still a lack of a gold standard to assess the nutritional status of patients, which is the purpose of our study of the GLIM criteria. In our study, the GLIM criteria divided preoperative patients into 3 groups: no malnutrition, moderate malnutrition (stage 1) and severe malnutrition (stage 2). According to the results of univariable and multivariable logistic regression (Table 2), compared with patients without malnutrition, malnutrition (moderate and severe) was associated with poor postoperative motor functional recovery, and there was a positive correlation between nutritional status and postoperative motor functional recovery. Thus, GLIM-defined malnutrition was demonstrated to have good discriminatory ability for predicting the motor functional recovery in older hip fracture patients.

The commonly used nutritional screening tools MNA-SF and NRS-2002 can also effectively predict postoperative motor function, which is consistent with previous results.^[35]^ Nevertheless, we constructed ROC and DCA curves according to the GLIM criteria, MNA-SF and NRS-2002 standards to demonstrate whether the GLIM criteria were better to predict postoperative motor functional recovery. Due to the AUC of the ROC, we found that the 3 screening tools all have predictive value. but the AUC of the GLIM criteria was greater than that of the MNA-SF and NRS-2002, indicating better predictability of the postoperative motor functional recovery. The calibration curve also showed that the predicted values were in good agreement with the observed results. More importantly, the DCA curve of the GLIM criteria showed a greater net benefit than the MNA-SF and NRS- 2002, indicating better clinical value. We established a nomograph model including the GLIM criteria to accurately predict the prognosis of older patients with hip fracture who are at risk of malnutrition, making it more intuitive and convenient to conduct individualized evaluation of patients in clinical work. Based on our experimental results, we believe that GLIM can better evaluate the nutritional status of hip fracture patients compared to MNA-SF and NRS-2002, and is a better nutritional assessment tool for predicting long-term motor functional recovery of the hip joint in postoperative patients.

The most important difference between the GLIM criteria and MNA-SF and NRS-2002 is that they use their own performance and etiological indicators to diagnose malnutrition in patients after screening with conventional screening tools, which may account for its more accurate diagnosis. BMI is a usual anthropometric assessment of nutritional status in older people.^[36]^ GLIM criteria, MNA-SF and NRS-2002 screening criteria all take into account the impact of BMI on nutritional status. High BMI in older patients shows the capability of achieving and storing energy, and patients with high BMI can provide the energy required for surgery and fracture healing. However, GLIM criteria not only classified according to BMI, but also have different evaluation criteria for patients aged < 70 and ≥ 70 years between moderate and severe malnutrition. Different age groups have different evaluation criteria for BMI. Meanwhile, the GLIM criteria incorporate muscle mass as an evaluation criterion. This may be why the GLIM criteria have a better screening effect, and this may account for the different predictive power of the 3 screening tools.

There were several limitations of this study. First, the number of patients was not sufficient, which may have affected the accuracy of the results. We should include a larger sample size for further research. Second, for muscle mass measurement in the GLIM criteria, the European Society for Clinical Nutrition and Metabolism recommends the use of the fat free mass index for the diagnosis of malnutrition.^[37]^ The European Working Group on Sarcopenia in Old People recommends using limb skeletal muscle mass and limb skeletal muscle index to evaluate muscle mass.^[38]^ The skeletal muscle mass index (SMI) is a commonly used indicator for evaluating muscle quality in included studies.^[23]^ But there was no recognized indicator of muscle loss in the GLIM criteria, and there was no accurate measurement method, so the selection of muscle mass may cause some bias in the analysis results. This requires future more samples research to verify the effectiveness of different muscle mass assessment methods in GLIM criteria.

## 5. Conclusion

The GLIM criteria can be used to diagnose preoperative malnutrition and predict prognosis of hip fracture in older patients. Compared with NRS-2002 and MNA-SF, the GLIM criteria for nutritional assessment demonstrated better predictive value for the recovery of postoperative hip joint motor function 1 year after surgery. Hence, for older hip fracture patients with GLIM-defined preoperative malnutrition, standardized nutritional treatment appear to be particularly important for the recovery of motor function and reduction of postoperative dysfunction.

## Author contributions

**Conceptualization:** Weicheng Wu, Zhening Guo, Zenghui Gu, Yongtao Mao, Chang She, Jun Gu, Bo Lv, Wei Xu, Liubing Li.

**Funding acquisition:** Liubing Li.

**Methodology:** Weicheng Wu, Zhening Guo.

**Project administration:** Wei Xu, Liubing Li.

**Supervision:** Wei Xu, Liubing Li.

**Writing – original draft:** Weicheng Wu, Zhening Guo.

**Writing – review & editing:** Weicheng Wu, Zhening Guo, Zenghui Gu, Yongtao Mao, Chang She, Jun Gu, Bo Lv, Wei Xu, Liubing Li.

## References

[1] Veronese N Maggi S. Epidemiology and social costs of hip fracture. Injury. 2018;49:1458–60. 29699731 10.1016/j.injury.2018.04.015

[2] Malafarina V Reginster JY Cabrerizo S. Nutritional status and nutritional treatment are related to outcomes and mortality in older adults with hip fracture. Nutrients. 2018;10:555. 29710860 10.3390/nu10050555 PMC5986435

[3] Vosoughi AR Emami MJ Pourabbas B. Factors increasing mortality of the elderly following hip fracture surgery: role of body mass index, age, and smoking. Musculoskelet Surg. 2017;101:25–9. 27766497 10.1007/s12306-016-0432-1

[4] Valizadeh M Mazloomzadeh S Golmohammadi S. Mortality after low trauma hip fracture: a prospective cohort study. BMC Musculoskelet Disord. 2012;13:143. 22883372 10.1186/1471-2474-13-143 PMC3512527

[5] Ito Y Wakabayashi H Nishioka S. Impact of rehabilitation dose on nutritional status at discharge from a convalescent rehabilitation ward in malnourished patients with hip fracture. Healthcare (Basel). 2021;9:722. 34204642 10.3390/healthcare9060722 PMC8231257

[6] Inoue T Misu S Tanaka T. Pre-fracture nutritional status is predictive of functional status at discharge during the acute phase with hip fracture patients: a multicenter prospective cohort study. Clin Nutr. 2017;36:1320–5. 27612921 10.1016/j.clnu.2016.08.021

[7] Goisser S Schrader E Singler K. Malnutrition according to mini nutritional assessment is associated with severe functional impairment in geriatric patients before and up to 6 months after hip fracture. J Am Med Dir Assoc. 2015;16:661–7. 25864084 10.1016/j.jamda.2015.03.002

[8] Malafarina V Uriz-Otano F Malafarina C. Effectiveness of nutritional supplementation on sarcopenia and recovery in hip fracture patients. A multi-centre randomized trial. Maturitas. 2017;101:42–50. 28539168 10.1016/j.maturitas.2017.04.010

[9] Sim SD Sim YE Tay K. Preoperative hypoalbuminemia: Poor functional outcomes and quality of life after hip fracture surgery. Bone. 2021;143:115567. 32745690 10.1016/j.bone.2020.115567

[10] White JV Guenter P Jensen G. Consensus statement: Academy of Nutrition and Dietetics and American Society for Parenteral and Enteral Nutrition: characteristics recommended for the identification and documentation of adult malnutrition (undernutrition). J Parenter Enteral Nutr. 2012;36:275–83. 10.1177/0148607112440285 22535923

[11] Mazzola P Ward L Zazzetta S. Association between preoperative malnutrition and postoperative delirium after hip fracture surgery in older adults. J Am Geriatr Soc. 2017;65:1222–8. 28263371 10.1111/jgs.14764 PMC6555399

[12] Inoue T Misu S Tanaka T. Acute phase nutritional screening tool associated with functional outcomes of hip fracture patients: a longitudinal study to compare MNA-SF, MUST, NRS-2002 and GNRI. Clin Nutr. 2019;38:220–6. 29456030 10.1016/j.clnu.2018.01.030

[13] Koren-Hakim T Weiss A Hershkovitz A. Comparing the adequacy of the MNA-SF, NRS-2002 and MUST nutritional tools in assessing malnutrition in hip fracture operated elderly patients. Clin Nutr. 2016;35:1053–8. 26231340 10.1016/j.clnu.2015.07.014

[14] Kondrup J Rasmussen HH Hamberg O. Nutritional risk screening (NRS 2002): a new method based on an analysis of controlled clinical trials. Clin Nutr. 2003;22:321–36. 12765673 10.1016/s0261-5614(02)00214-5

[15] Baek MH Heo YR. Evaluation of the efficacy of nutritional screening tools to predict malnutrition in the elderly at a geriatric care hospital. Nutr Res Pract. 2015;9:637–43. 26634053 10.4162/nrp.2015.9.6.637 PMC4667205

[16] Huang DD Wu GF Luo X. Value of muscle quality, strength and gait speed in supporting the predictive power of GLIM-defined malnutrition for postoperative outcomes in overweight patients with gastric cancer. Clin Nutr. 2021;40:4201–8. 33583658 10.1016/j.clnu.2021.01.038

[17] Li Y Peng Z Xu D. The GLIM criteria represent a more appropriate tool for nutritional assessment in patients with Crohn’s disease. Front Nutr. 2022;9:826028. 35419396 10.3389/fnut.2022.826028 PMC9000965

[18] Zhang X Tang M Zhang Q. The GLIM criteria as an effective tool for nutrition assessment and survival prediction in older adult cancer patients. Clin Nutr. 2021;40:1224–32. 32826109 10.1016/j.clnu.2020.08.004

[19] Kootaka Y Kamiya K Hamazaki N. The GLIM criteria for defining malnutrition can predict physical function and prognosis in patients with cardiovascular disease. Clin Nutr. 2021;40:146–52. 32571679 10.1016/j.clnu.2020.04.038

[20] Phruetthiphat OA Pinijprapa P Satravaha Y. An innovative scoring system for predicting an excellent Harris hip score after proximal femoral nail anti-rotation in elderly patients with intertrochanteric fracture. Sci Rep. 2022;12:19939. 36402794 10.1038/s41598-022-24177-7 PMC9675850

[21] Trollebø MA Skeie E Revheim I. Comparison of nutritional risk screening with NRS2002 and the GLIM diagnostic criteria for malnutrition in hospitalized patients. Sci Rep. 2022;12:19743. 36396666 10.1038/s41598-022-23878-3 PMC9672100

[22] Jensen GL Cederholm T Correia M. GLIM criteria for the diagnosis of malnutrition: a consensus report from the global clinical nutrition community. JPEN J Parenter Enteral Nutr. 2019;43:32–40. 30175461 10.1002/jpen.1440

[23] Yang M Hu X Wang H. Sarcopenia predicts readmission and mortality in elderly patients in acute care wards: a prospective study. J Cachexia Sarcopenia Muscle. 2017;8:251–8. 27896949 10.1002/jcsm.12163 PMC5377397

[24] Xie HL Ruan GT Wei L. The prognostic value of the combination of body composition and systemic inflammation in patients with cancer cachexia. J Cachexia Sarcopenia Muscle. 2023;14:879–90. 36872512 10.1002/jcsm.13205 PMC10067477

[25] Shimizu A Fujishima I Maeda K. Effect of low tongue pressure on nutritional status and improvement of swallowing function in sarcopenic dysphagia. Nutrition. 2021;90:111295. 34107332 10.1016/j.nut.2021.111295

[26] Chen LK Liu LK Woo J. Sarcopenia in Asia: consensus report of the Asian Working Group for Sarcopenia. J Am Med Dir Assoc. 2014;15:95–101. 24461239 10.1016/j.jamda.2013.11.025

[27] Cederholm T Barazzoni R Austin P. ESPEN guidelines on definitions and terminology of clinical nutrition. Clin Nutr. 2017;36:49–64. 27642056 10.1016/j.clnu.2016.09.004

[28] Fiorindi C Dragoni G Scaringi S. Relationship between nutritional screening tools and GLIM in complicated IBD requiring surgery. Nutrients. 2021;13:3899. 34836154 10.3390/nu13113899 PMC8623109

[29] Gascón-Ruiz M Casas-Deza D Torres-Ramón I. GLIM vs ESPEN criteria for the diagnosis of early malnutrition in oncological outpatients. Clin Nutr. 2021;40:3741–7. 34130019 10.1016/j.clnu.2021.04.025

[30] Inoue T Maeda K Nagano A. Undernutrition, sarcopenia, and frailty in fragility hip fracture: advanced strategies for improving clinical outcomes. Nutrients. 2020;12:3743. 33291800 10.3390/nu12123743 PMC7762043

[31] Semel J Gray JM Ahn HJ. Predictors of outcome following hip fracture rehabilitation. Pm r. 2010;2:799–805. 20869677 10.1016/j.pmrj.2010.04.019

[32] Li HJ Cheng HS Liang J. Functional recovery of older people with hip fracture: does malnutrition make a difference? J Adv Nurs. 2013;69:1691–703. 23057761 10.1111/jan.12027

[33] Miu KYD Lam PS. Effects of nutritional status on 6-month outcome of hip fractures in elderly patients. Ann Rehabil Med. 2017;41:1005–12. 29354577 10.5535/arm.2017.41.6.1005 PMC5773420

[34] Nishioka S Wakabayashi H Momosaki R. Nutritional status changes and activities of daily living after hip fracture in convalescent rehabilitation units: a Retrospective Observational Cohort Study from the Japan Rehabilitation Nutrition Database. J Acad Nutr Diet. 2018;118:1270–6. 29752190 10.1016/j.jand.2018.02.012

[35] Gumieiro DN Rafacho BP Gonçalves AF. Mini Nutritional Assessment predicts gait status and mortality 6 months after hip fracture. Br J Nutr. 2013;109:1657–61. 23017491 10.1017/S0007114512003686

[36] Flodin L Laurin A Lökk J. Increased 1-year survival and discharge to independent living in overweight hip fracture patients: a prospective study of 843 patients. Acta Orthop. 2016;87:146–51. 26986549 10.3109/17453674.2015.1125282 PMC4812076

[37] Cederholm T Bosaeus I Barazzoni R. Diagnostic criteria for malnutrition – an ESPEN Consensus Statement. Clin Nutr. 2015;34:335–40. 25799486 10.1016/j.clnu.2015.03.001

[38] Cruz-Jentoft AJ Bahat G Bauer J. Sarcopenia: revised European consensus on definition and diagnosis. Age Ageing. 2019;48:601. 10.1093/ageing/afz046 PMC6593317 31081853

